# The impact of *Codonopsis Pilosulae* and *Astragalus Membranaceus* extract on growth performance, immunity function, antioxidant capacity and intestinal development of weaned piglets

**DOI:** 10.3389/fvets.2024.1470158

**Published:** 2024-09-23

**Authors:** Rongxia Guo, Hao Zhang, Chenghui Jiang, Chun Niu, Baoxia Chen, Ziwen Yuan, Yanming Wei, Yongli Hua

**Affiliations:** Institute of Traditional Chinese Veterinary Medicine, College of Veterinary Medicine, Gansu Agricultural University, Lanzhou, China

**Keywords:** *Codonopsis pilosulae* and *Astragalus membranaceus* extract, weaned piglets, growth performance, antioxidant capacity, immune performance, intestinal development

## Abstract

**Introduction:**

The objective of this study was to examine the impact of *Codonopsis pilosula* and *Astragalus membranaceus* extract (CA) on the growth performance, diarrhea rate, immune function, antioxidant capacity, gut microbiota, and short-chain fatty acids (SCFAs) in weaned piglets.

**Methods:**

A total of forty-eight 31-day-old weaned piglets, were divided into four groups randomly based on the treatment type: control group (CON), low dose group (LCA, 0.5% CA), medium dose group (MCA, 1.0% CA), and high dose group (HCA, 1.5% CA), and were fed for a duration of 28 days. On the morning of the 1st and 29th day, the piglets were assessed by weighing them on an empty stomach, recording their daily feed intake and diarrhea rate.

**Results:**

CA increased the average daily weight gain and reduced F/G without significant differences, and the diarrhea rate was reduced in the LCA and MCA groups. Furthermore, the levels of T-AOC, SOD, GSH-Px, and MDA were increased. The levels of T-AOC in the LCA group and the MCA group, SOD in the MCA group, and GSH-Px in the HCA group were significantly higher compared with the CON group (*p* < 0.05). Additionally, CA significantly increased IgM, IgG, and IgA levels (*p* < 0.05). The results of gut microbiota analysis showed that the bacterial population and diversity of faeces were changed with the addition of CA to basal diets. CA increased the abundance of the beneficial bacterial Firmicutes and Lactobacillus. Additionally, Compared with the CON group, CA significantly increased the SCFAs content of weaned piglets (*p* < 0.05).

**Discussion:**

CA can alleviate oxidative stress, improve immunity and antioxidant capacity, increase the abundance of beneficial bacteria, and the content of SCFAs for improving the intestinal barrier of piglets, thus promoting growth and reducing diarrhea rate in weaned piglets.

## 1 Introduction

Weaning of piglets is a necessary stage in the growth period of the pig and has a great impact on later performance (1). During this period, weaning causes stress in piglets, which increases the level of inflammatory factors in the body, activates the immune system, destroys the intestinal morphology and structure, and impairs the intestinal barrier function (2). This results in a decrease in the feed conversion rate, a slowdown in the growth rate, and the invasion of pathogens into the organism leading to an increase in the rate of diarrhea and mortality in piglets (3). Therefore, how to alleviate piglet weaning stress, promote the development of intestinal morphology and structure, improve intestinal barrier function, and enhance piglet production performance is a vital area of animal nutrition research (4). For a long time, antibiotics have been widely used as feed additives to resist the invasion of pathogens into the animal body and to improve the quality of livestock products (5). However, with antibiotic residues in animal products and the emergence of antibiotic-resistant bacteria, the use of antibiotics in animal husbandry has become an event of public concern (6, 7), Therefore countries around the world are gradually banning the use of antibiotics in animal feed and restricting the use of antibiotics in the animal breeding process (8) It is thus urgent to find a substitute for antibiotics in feed. Herbal medicines are one of the effective potential alternatives to antibiotics and are receiving increasing attention (9).

*Codonopsis pilosula* has the effect of invigorating the spleen to benefit the lung, promoting the production of body fluid. *Astragalus membranaceus* has the effects of invigorating vital energy, fixing the epidermal surface and stopping sweating, inducing diuresis to reduce oedema, generating fluids and nourishing the blood, promoting pus damage. *Codonopsis pilosulae* and *Astragalus membranaceus* are both important medicines for sustaining the healthy energy to eliminate evils, and their main components are polysaccharides, a variety of amino acids and trace elements, etc. (10, 11). Research has shown that a mixture of *Codonopsis pilosula* and *Astragalus membranaceus* can improve the ability of finishing pigs to resist high temperature and cold, promote growth, and improve the yield and quality of livestock products (12). The preclinical toxicological safety evaluation showed that the oral solution of Codonopsis polysaccharide is safe for long-term administration without obvious side effects, and has a good safety of medication (13). Addition of *Astragalus* polysaccharides to the basic diet improves growth performance, reduces diarrhea rate and improves immune function of weaned piglets (14). Although the anti-inflammatory and immunomodulatory effects of the polysaccharide components in *Codonopsis pilosulae* and *Astragalus membranaceus* have been well recognized, previous studies have focused on the regulation of immune function *in vitro* and in experimental animals, etc., and studies on the effects of CA on weaned piglets have not been reported. In this study, we assessed the effects of CA on the growth performance, immunity, antioxidant level, gut microbiota and SCFAs of weaned piglets, which can provide a reference for the rational application of the CA for feeding in pig production.

## 2 Materials and methods

### 2.1 Experimental animals

48 healthy weaned piglets (Duroc × Landrace × Large White), aged 31 days, were obtained from Gansu Jindi Herding Co. The experiment followed animal welfare regulations and was approved by the Animal Ethics Committee of Gansu Agricultural University.

### 2.2 Major instruments

The equipment used for the experiment includes a rotary evaporator (RE-6000) from Shanghai Yarong Biochemical Instrument Factory, a full-wavelength enzyme labeller (FK-058) from Beijing Putian Xinqiao Technology Co, an ultraviolet spectrophotometer (725 N) from Shanghai Yuanxi Instrument Co, a gas chromatography machine from Agilent Technologies Co, a freeze dryer from Beijing Bomikang Experimental Instrument Co, an automatic thermostatic electric heating jacket (ZHT-I) from Shandong Province Jancheng County Yongxing Instrument Factory, a vortex mixer from Haimen Qilinbel Instrument Manufacturing Co, and a pulveriser from Tianjin Tester Instrument Co.

### 2.3 Preparation of CA

*Codonopsis pilosula* and *Astragalus membranaceus* are dried herbs, purchased from Yellow River Herb Market, Lanzhou City, Gansu Province, and were stored in the 612 laboratory, Zhi Zhi Building, Gansu Agricultural University. The authentication of the herbs was carried out by Prof. Wei Yanming, Department of Chinese Veterinary Medicine, College of Animal Medicine, Gansu Agricultural University. The herbs were then roughly crushed using a pulveriser and decocted by adding 10 times the volume of distilled water at 1:1 for 1 h. Decoction was carried out twice, and then the filtrate was added to 10 times the volume of ethanol. This was followed by another decoction, after which it was mixed with the filtrate three times and concentrated using a rotary evaporator. The subsequent step involved freeze-drying in a freeze-dryer to obtain *CA.* The polysaccharide content of CA was found to be 65.2%, and the flavonoid content was 0.172 mg/g, as analyzed by ultraviolet spectrophotometer.

### 2.4 Animals and experimental design

Forty-eight 31-day-old healthy weaned piglets, ear-tagged and numbered, were randomly divided into four groups according to body weight, with males and females in each group evenly distributed: CON (basal diet), LCA (basal diet +0.5%CA), MCA (basal diet +1.0%CA), and HCA (basal diet +1.5%CA). Each group of piglets was housed in a pen (4 m × 3 m × 1.0 m) with a concrete leaky floor and an ambient temperature of 33 ± 2°C, and disinfected once a day in the morning. After a 2-day adaptation period, the experimental period lasted for 28 days. During the experimental period, the feeding management followed routine pig farm procedures, feeding was carried out 5 times a day, and the bottom of the trough was used as the standard for each feeding, ensuring no residual material before the next feeding. Each group of piglets was housed in a pen (4 m × 3 m × 1.0 m) with a concrete leaky floor and an ambient temperature of 33 ± 2°C, and disinfected once a day in the morning. Additionally, the piglets were provided with free access to food and water, and their food intake was recorded daily. The composition and nutrient level of the basal diet (dry matter basis) are shown in Table 1.

**Table 1:** Ingredient composition and nutrient levels of diets (dry matter basis).

Ingredients	Content (%)	Nutrient levels	Content (%)
Corn	54	Crude protein (CP)	19.0
Fishmeal	1.0	Crude fiber (CF)	5.0
Soybeans	4.9	Lysine	1.3
Soybean meal	25.3	Methionine	0.3
Wheat	2.0	Threonine	0.75
Wheat bran	5.5	Ca	0.6
Yeast	1.0	Total phosphorus (TP)	0.5
Soybean oil	0.5	Ash	7.0
Ca(H_2_PO4)_2_	1.2		
Talcum powder	1.1		
NaCl	0.5		
Total	100		

### 2.5 Sample collection and processing

Fresh faeces were collected from each piglet under aseptic conditions on the 29th day of the experiment and stored frozen at −80°C. Blood was collected from the anterior vena cava for each test group and placed in a capped 10 mL centrifuge tube. The blood samples were then centrifuged at 4,000 r/min for 15 min to separate the serum, which was stored frozen at −80°C.

### 2.6 Indicator measurement

#### 2.6.1 Growth performance and diarrhea rate

Each piglet was weighed on the mornings of the 1st and 29th day of the trial. Feed intake was recorded daily to calculate the average daily gain (ADG), average daily feed intake (ADFI), and feed conversion ratio (F/G). Incidences of diarrhea among the piglets were also recorded during the trial. The attributed score for diarrhea was as follows: 0, normal; 1, loose stool; 2, loose/some diarrhea; 3, diarrhea; 4, severe watery diarrhea. Diarrhea rate was calculated according to the following formula: diarrhea rate (%) = (number of piglets with diarrhea*diarrhea days)/ (number of piglets*total observational days) *100 (15).

#### 2.6.2 Serum antioxidant indices

The GSH-Px, MDA, SOD, and T-AOC kits from Jianchen in Nanjing, China were used to measure antioxidant levels in the serum. The information of the kits is presented in Table 2.

**Table 2:** Serum antioxidant index kits and their product numbers.

Items	Kit names	Product numbers
GSH-Px	Glutathione Peroxidase (GSH-Px) assay kit (Colorimetric method)	A005-1-2
MDA	Malondialdehyde (MDA) assay kit (TBA method)	A003-1-2
SOD	Superoxide Dismutase (SOD) assay kit (WST-1method)	A001-3-1
T-AOC	Total antioxidant capacity assay kit	A015-2-1

#### 2.6.3 Serum immunoglobulin levels

Serum immunoglobulin levels, including IgA, IgG, and IgM, were determined using ELISA kits in accordance with the kit instructions. The information of the kits is presented in Table 3.

**Table 3:** Serum immune index kits and their product numbers.

Items	Kit names	Product numbers
IgA	Immunoglobulin A assay kit	F8212-A
IgG	Immunoglobulin G assay kit	F4555-A
IgM	Immunoglobulin M assay kit	F4554-A

#### 2.6.4 Gut microbiota analysis

Bacterial DNA was extracted from piglet fecal samples using a kit. The V3 + V4 regions of 16SrRNA genes were amplified by PCR with a pair of universal primers (Forward: ACTCCTACGGGAGGCAGCA, Reverse: GGACTACHVGGGTWTCTAAT) tagged with barcodes to produce the fragments of about 500 bp with DNA polymerase. The quality and concentration of microbial genes were examined on 1.2% agarose gel. The PCR products were checked and purified with 2% agarose gel electrophoresis. The amplification products were recovered by magnetic beads, quantified by fluorescence with a Microplate reader, and each sample was mixed in proportion to the required volume. The Illumina NovaSeq platform performed Misep sequencing on all libraries. Data quality control was performed, removing low quality sequences and chimeric sequences to ensure data accuracy. Sequences were then categorized into different OUTs according to 97% similarity to form OUT clusters. Representative sequences were selected to compare with the database for species identification. Subsequently, the α-diversity index, β-diversity index, and intergroup differences were analyzed.

#### 2.6.5 SCFAs analysis

##### 2.6.5.1 GC conditions

The Aligent J&W GC column (DB-FFAP, 30 mm × 0.25 mm, 0.25 μm) was used in the chromatographic analysis. The temperature of the injection port was 220°C. The column temperature increase procedure consisted of the following steps: the initial temperature was 60°C and maintained for 2 min, then it was increased to 120°C at a rate of 10°C/min and maintained for 2 min, and finally, it was increased to 180°C at a rate of 15°C/min and maintained for 5 min. The samples were determined using the shunt method with a shunt ratio of 20:1 and a manual injection volume of 1 μL. High-purity nitrogen (purity greater than 99%) was used as the carrier gas at a flow rate of 1 mL/min. The flame ionisation detector (FID) was employed at a temperature of 280°C.

##### 2.6.5.2 Establishment of standard curves

First, six standards of acetic acid (90 μL), propionic acid (50 μL), n-butyric acid (10 μL), isobutyric acid (40 μL), n-valeric acid (20 μL), and isovaleric acid (20 μL) were precisely measured at the volume ratio of 9: 5:1:4:2:2. The six standard solutions were then mixed well and diluted with ultrapure water to obtain six mixed standard solutions with different concentrations (5,000-fold, 4,000-fold, 3,000-fold, 2,000-fold, 1,000-fold, and 500-fold). N-butanol was drawn up in 20 μL and 980 μL of ultrapure water was added to make an n-butanol dilution. For example, 5,000-fold was used, and 1 μL of the mixed standard solution was aspirated. Subsequently, 4,949 μL of ultrapure water and 50 μL of the internal standard diluent were added to achieve a sampling concentration of N-butanol at 1.079 mmol/L. Next, 1 μL of the sample was accurately aspirated and detected on the machine with N-butanol as the internal standard. Finally, the standard curve was obtained with the content ratio as the horizontal coordinate and the peak height ratio as the vertical coordinate.

##### 2.6.5.3 Preparation of faecal sample solutions

Faecal samples weighing 0.20 g were accurately placed in 2 mL EP tubes. Then, four times the volume of ultrapure water was added, mixed well, and allowed to stand at room temperature for 20 min. After this, the mixture was centrifuged at 15000 r/min and 4°C for 15 min. The resulting supernatant was aspirated into 2 mL EP tubes, while the faecal precipitates were subjected to the same procedure with four times the volume of ultrapure water. The combined supernatant from both operations was then centrifuged again, and the resulting supernatant was aspirated into 990 μL. Next, 10 μL of n-butanol dilution solution was added, mixed well, and the mixture was filtered through a 0.22 μm membrane. Finally, 1 μL was accurately pipetted for GC detection (16).

##### 2.6.5.4 Repeatable experiments

Six parallel samples were prepared using the method described in “2.6.5.3.” Then, the retention times and peak areas of the six components were detected following the chromatographic conditions in “2.6.5.1.” Subsequently, RSD values were calculated and reproducibility was verified.

##### 2.6.5.5 Stability experiment

One sample was prepared according to the processing method described in “2.6.5.3,” and the samples were injected at six different time intervals according to the chromatographic conditions described in “2.6.5.1.” Subsequently, the retention times and peak areas of the six components were detected, and the RSD values were calculated.

##### 2.6.5.6 Precision experiments

Based on the chromatographic conditions specified in “2.6.5.1,” we performed six injections of a 500-fold diluted mixed standard solution into the sample. We then detected the retention time and peak area of the six components and calculated their respective RSD values.

##### 2.6.5.7 Reference standard recovery experiment

Samples prepared following the treatment in Section “2.6.5.3” are spiked with a standard mixture of known concentration, and the spiked recoveries are then calculated based on the chromatographic conditions in Section “2.6.5.1.”

##### 2.6.5.8 Determination of SCFAs in faecal samples

The processing method described in “2.6.5.3” involved preparing six samples for each group and detecting the content of the six components using GC assay. Specifically, 1 μL of the samples was accurately aspirated according to the chromatographic conditions in “2.6.5.1.”

## 3 Statistics analysis

The data were presented as mean ± standard error of the mean. Experimental data were analyzed using one-way ANOVA in SPSS Statistics 26.0 software, with statistical significance defined as *p* < 0.05. Plots were created using GraphPad Prism 8 software.

## 4 Results

### 4.1 Effects of CA on growth performance and diarrhea rate in weaned piglets

Based on the results in Table 4, there was no significant difference in IBW among the three groups. The addition of CA to the basal diet resulted in an increasing trend in body weights of the weaned piglets, with the MCA group exhibiting the highest ADG and the lowest F/G. Moreover, the diarrhea rates in the LCA and MCA groups were reduced by 12.5 and 50.0% compared to the CON group.

**Table 4:** Effects of CA on growth performance and diarrhea rate of weaned piglets.

Item	CON	LCA	MCA	HCA	*p* value
IBW (kg)	9.06 ± 1.68	8.63 ± 1.20	9.05 ± 1.71	9.16 ± 2.08	0.62
FBW (kg)	16.76 ± 368	16.65 ± 2.10	19.05 ± 3.09	17.00 ± 3.93	0.25
TG (kg)	7.70 ± 2.11	8.01 ± 1.22	10.00 ± 3.57*	7.83 ± 2.12	0.05
ADG (g/d)	296.1 ± 81.5	308.3 ± 47.0	384.6 ± 60.6*	301.2 ± 81.7	0.05
ADFI (g/d)	586.60	479.25	568.91	580.27	–
F/G	1.98	1.55	1.47	1.92	–
Diarrhea rate (%)	5.12	4.48	2.56	6.41	–

CON group (piglets fed basal diet); LCA group (piglets fed basal diet + 0.5% CA); MCA group (piglets fed basal diet + 1.0% CA); HCA group (piglets fed basal diet + 1.5% CA).*Indicates statistical differences between groups: *p < 0.05 indicates significant differences; **p < 0.01 indicates highly significant differences.

### 4.2 Effects of CA on serum antioxidant levels in weaned piglets

**Figure 1:**
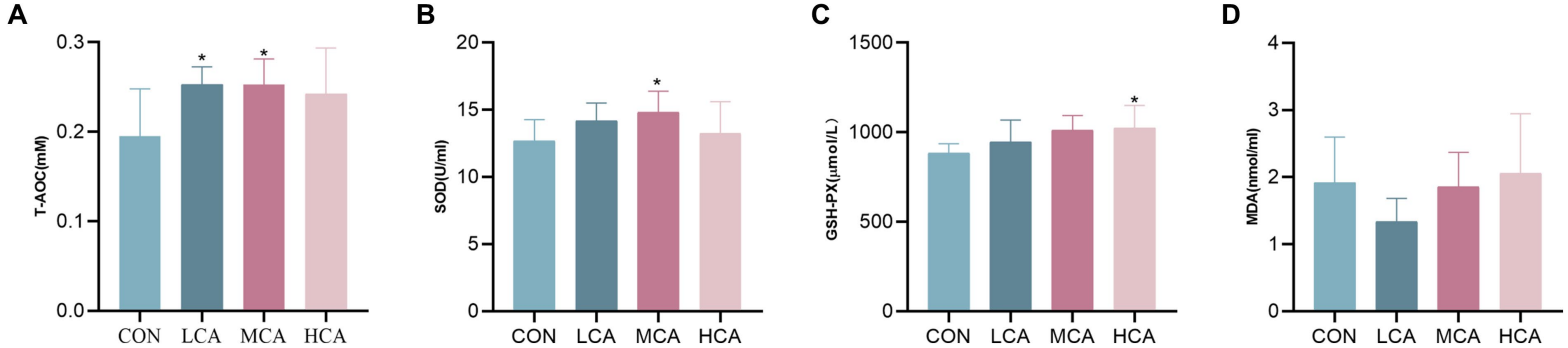
Effects of CA on serum antioxidant levels of weaned piglets. **(A)** Serum T-AOC level. **(B)** Serum SOD level. **(C)** Serum GSH-px level. **(D)** Serum MDA level. CON group (piglets fed basal diet). LCA group (piglets fed basal diet +0.5% CA). MCA group (piglets fed basal diet +1.0% CA). HCA group (piglets fed basal diet +1.5% CA). Mean ± SEM are shown (*n* = 8). *Indicates statistical differences between groups: *p < 0.05 indicates significant differences; **p < 0.01 indicates highly significant differences.

The activity of T-AOC in the LCA and MCA groups, and the activity of GSH-Px in the HCA group were significantly higher (*p* < 0.05) compared to the CON group, as demonstrated in Figure 1. However, there was no significant difference in serum MDA activity in the LCA, MCA, and HCA groups compared to the CON group (*p* > 0.05). Additionally, the activity of SOD in the MCA group was also significantly higher than the CON group.

### 4.3 Effects of CA on serum immunity level of weaned piglets

**Figure 2:**
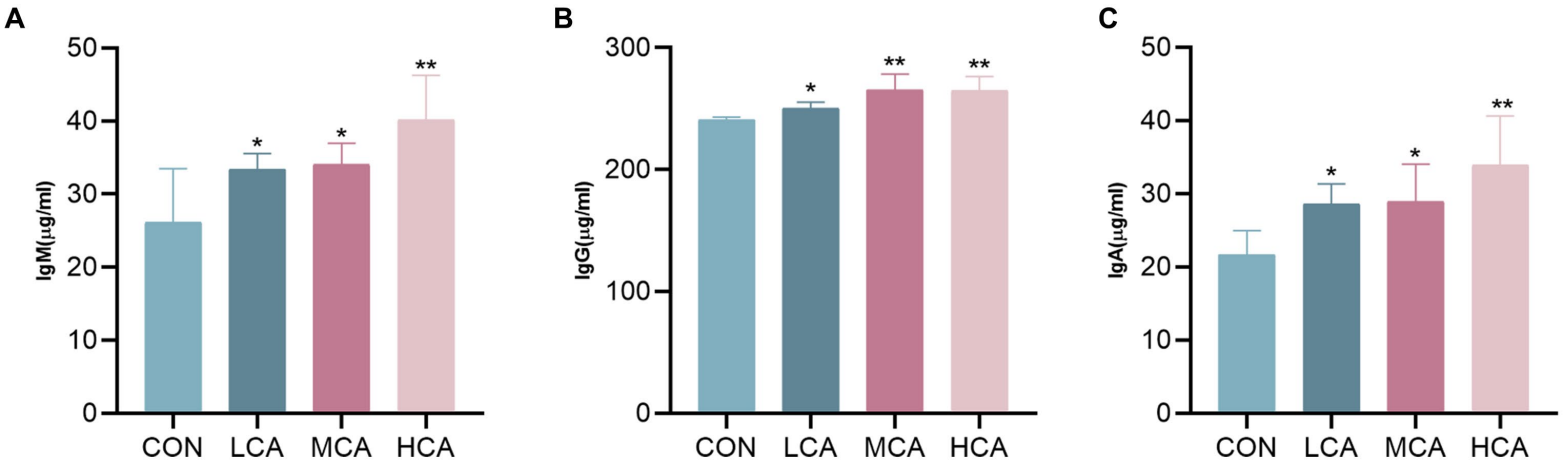
Effects of CA on serum immunoglobulin of weaned piglets. **(A)** Serum IgM level. **(B)** Serum IgG level. **(C)** Serum IgA level. CON group (piglets fed basal diet). LCA group (piglets fed basal diet +0.5% CA). MCA group (piglets fed basal diet +1.0% CA). HCA group (piglets fed basal diet +1.5% CA). Mean ± SEM are shown (*n* = 8). ^*^Indicates statistical differences between groups: ^*^p < 0.05 indicates significant differences; ^**^p < 0.01 indicates highly significant differences.

The levels of IgM, IgG, and IgA were significantly higher in the LCA, MCA, and HCA groups compared to the CON group (*p* < 0.05, Figure 2), suggesting that CA could increase the immunoglobulin levels of weaned piglets and enhance their immunity.

### 4.4 Effects of CA on gut microbiota of weaned piglets

**Figure 3:**
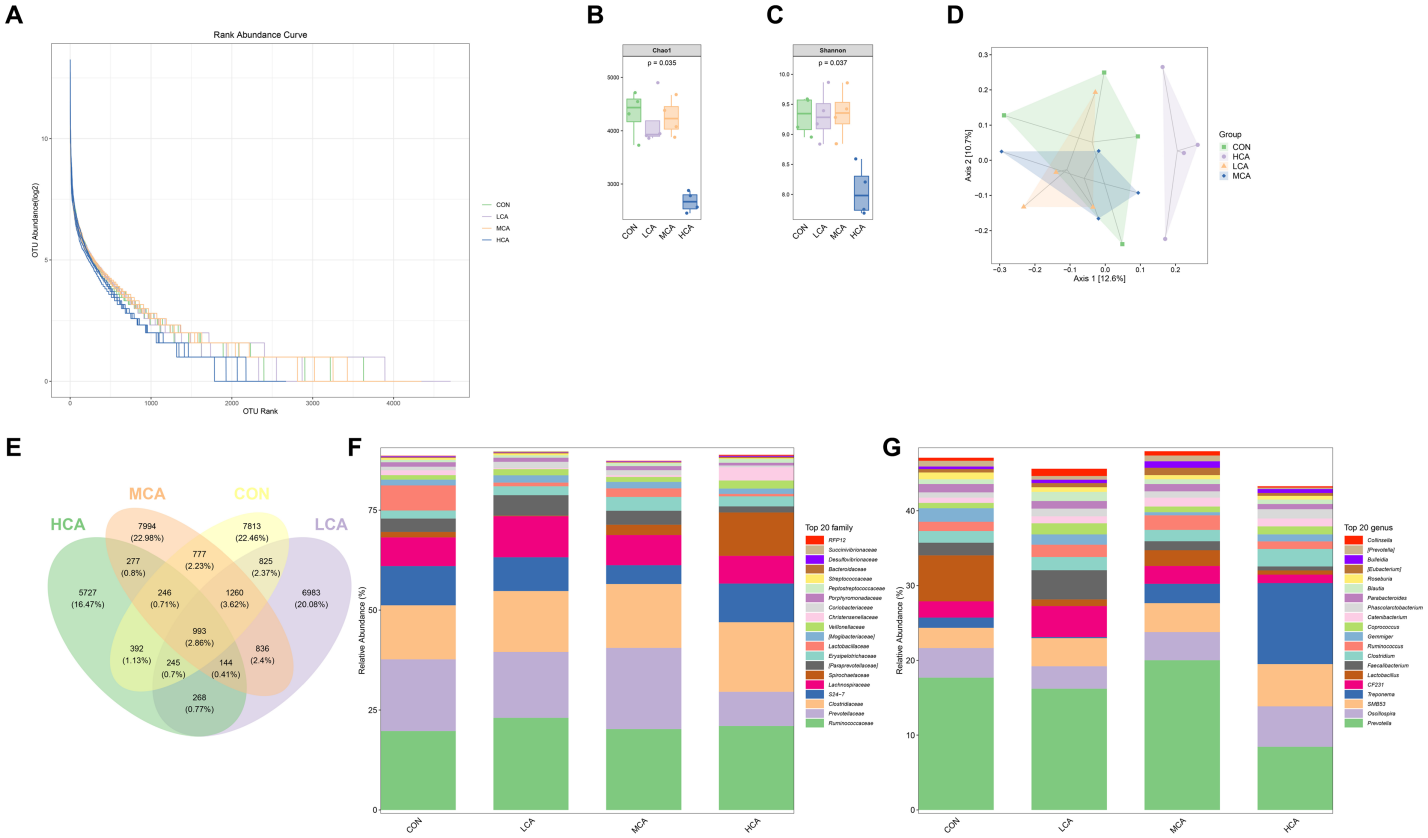
Effects of CA on the faecal-like flora of weaned piglets **(A)** SPecaccum species accumulation plot. **(B,C)** Grouped box plots of the AlPha diversity index. **(D)** Two-dimensional ordination plot of samples analysed by Beta Diversity PCoA. **(E)** Venn diagram of ASV/OUT. **(F)** Distribution of taxonomic composition at the Phyla level. **(G)** Distribution of taxonomic composition at the genus level. CON group (piglets fed basal diet). LCA group (piglets fed basal diet +0.5% CA). MCA group (piglets fed basal diet +1.0% CA). HCA group (piglets fed basal diet +1.5% CA). Mean ± SEM are shown (*n* = 4).

High-throughput sequencing of 16SrRNA was used to describe changes in the gut microbiota of weaned piglets. The species accumulation curve of the SPecaccum was found to be flat, indicating good homogeneity in bacterial community composition and minimal differences in abundance among ASVs/OTUs, which met the sequencing requirements (Figure 3A). The Chao1 index, used to measure community abundance, revealed that the CON group had the highest community richness, followed by the MCA, LCA, and HCA groups. Notably, the Chao1 index was significantly lower (*p* < 0.05) in the HCA group compared to the CON group, indicating lower community richness in the HCA group. Furthermore, the assessment of community diversity using the Shannon index showed that the MCA group had the highest diversity, followed by the CON, LCA, and HCA groups (Figures 3B,C), Which suggests that the community diversity of the MCA group was greater compared to the other groups. Principal Coordinate Analysis (PCoA) demonstrated that the CON group was more similar to the microbiota of the LCA and MCA groups, but different from that of the HCA group, showing good intra-group reproducibility (Figure 3D). The composition of the gut microbiota is illustrated in the Venn diagram, indicating unique ASV/OTUs for the CON, LCA, MCA, and HCA groups as 7,813, 6,983, 7,994, and 5,727 respectively, with 993 ASV/OTUs common to all four groups (Figure 3E). The main bacterial phyla in piglet feces were Firmicutes, Bacteroidetes, Spirochaetes, Actinobacteria, Tenericutes, and Proteobacteria. CA increased the relative abundance of Firmicutes in weaned piglets compared with the CON group, while the relative abundance of Bacteroidetes was higher in the LCA and HCA groups, and Spirochaetes was elevated in the MCA and HCA groups (Figure 3F). At the genus level, the dominant genera in all samples were Clostridiaceae, Oscillospira, Treponema, Bacteroidales, Lachnospiraceae, Lactobacillus, Ruminococcaceae, and Faecalibacterium. Moreover, the relative abundance of Bacteroidales in the HCA group, Clostridiaceae in the HCA and MCA groups, Lactobacillus in the LCA and MCA groups, Prevotella in the MCA group, and Faecalibacterium in the MCA group were all elevated (Figure 3G).

### 4.5 Effects of CA on SCFAs in weaned piglets

#### 4.5.1 Establishment of a method for the determination of SCFAs

##### 4.5.1.1 Retention times and chromatographic peaks of SCFAs

**Figure 4:**
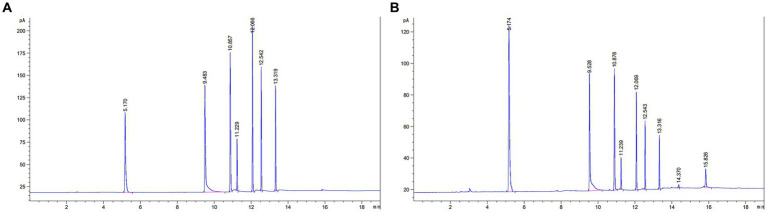
Chromatograms of SCFA. **(A)** SCFA chromatogram of mixed standards. **(B)** SCFA chromatogram of faecal samples. (1) N-butanol (internal marking); (2) Acetic acid; (3) ProPionic acid; (4) Isobutyric acid; (5) N-butyric acid; (6) Isovaleric acid; (7) N-valeric acid.

According to the chromatographic conditions of “2.6.5.1,” 1 μL of the mixed standard and sample solution were taken, respectively. The retention time results are shown in Table 5. As can be seen in Figure 4, the peaks of the components were well separated, the baseline was smooth, and the retention times of the standards and samples corresponded well.

**Table 5:** Retention times of the six SCFAs and n-butanol (internal standard).

Short-chain fatty acids	Retention time/min (standard)	Retention time/min (sample)
N-butanol	5.170	5.174
Acetic acid	9.483	9.528
ProPionic acid	10.857	10.878
Isobutyric acid	11.229	11.239
N-butyric acid	12.066	12.069
Isovaleric acid	12.542	12.543
N-valeric acid	13.319	13.316

##### 4.5.1.2 Examination of linear relationships

The regression equations, linear ranges, and correlation coefficients of acetic acid, propionic acid, isobutyric acid, n-butyric acid, isovaleric acid, and n-valeric acid were obtained according to the chromatographic conditions of “2.6.5.1,” with the ratio of the content as the horizontal coordinate (X) and the ratio of the peak height as the vertical coordinate (Y). The mixed standard solution was diluted into different gradients of mixed standards, and the concentration of individual standards at each shaving was calculated. In Table 6, the substances showed good linear relationships with correlation coefficients greater than 0.99.

**Table 6:** Regression equation and linear range determination of six SCFAs.

Short-chain fatty acids	Regression equation	Linear range	Correlation coefficient
Acetic acid	Y = 0.988696X + 0.133356	1.3878 ~ 13.8777	0.99548
ProPionic acid	Y = 0.290642X + 0.0483997	0.5917 ~ 5.9166	0.99882
Isobutyric acid	Y = 0.668053X + 0.0206236	0.0951 ~ 0.9507	0.99857
N-butyric acid	Y = 0.526346X + 0.0601459	0.3842 ~ 3.8424	0.99880
Isovaleric acid	Y = 0.931860X + 0.0367352	0.1623 ~ 1.6223	0.99886
N-valeric acid	Y = 0.79096X + 0.0396164	0.1607 ~ 1.6074	0.99759

Y: peak height ratio; X: content ratio.

##### 4.5.1.3 Repeatability results

The RSD values of retention time for acetic acid, propionic acid, isobutyric acid, n-butyric acid, isovaleric acid, and n-pentanoic acid in the faecal samples were 0.06, 0.03, 0.01, 0.01, 0.006, and 0.008%. Similarly, the RSD values of peak heights were 12.33, 7.19, 0.76, 1.94, 3.00, and 2.47%, respectively. These results collectively indicate that the method is stable, reliable, and boasts good reproducibility.

##### 4.5.1.4 Stability results

The RSD values of retention time of acetic acid, propionic acid, isobutyric acid, n-butyric acid, isovaleric acid and n-pentanoic acid in the stool samples were 0.06, 0.03, 0.02, 0.01, 0.01, 0.008%. In addition, the RSD values of peak heights were 11.62, 5.47, 5.12, 6.51, 3.00, 6.01%. These values indicate that the solutions of feces samples exhibited good stability.

##### 4.5.1.5 Precision results

The precision of the assay was good, as indicated by the RSD values of the retention times of the mixed standards acetic acid, propionic acid, isobutyric acid, n-butyric acid, isovaleric acid and n-pentanoic acid, which were 0.06, 0.04, 0.02, 0.01, 0.01, 0.005%. Additionally, the RSD values of the peak heights were 10.74, 5.68, 3.55, 6.77, 5.59, and 6.47%, respectively.

##### 4.5.1.6 Spiking recovery test

The recoveries of acetic acid, propionic acid, isobutyric acid, n-butyric acid, isovaleric acid, and n-pentanoic acid were 90.79, 122.94, 93.41, 95.64, 96.22, and 93.06%, respectively. These results suggest that the assay recoveries were good.

#### 4.5.2 Effects of CA on the content of SCFAs in weaned piglets

**Figure 5:**
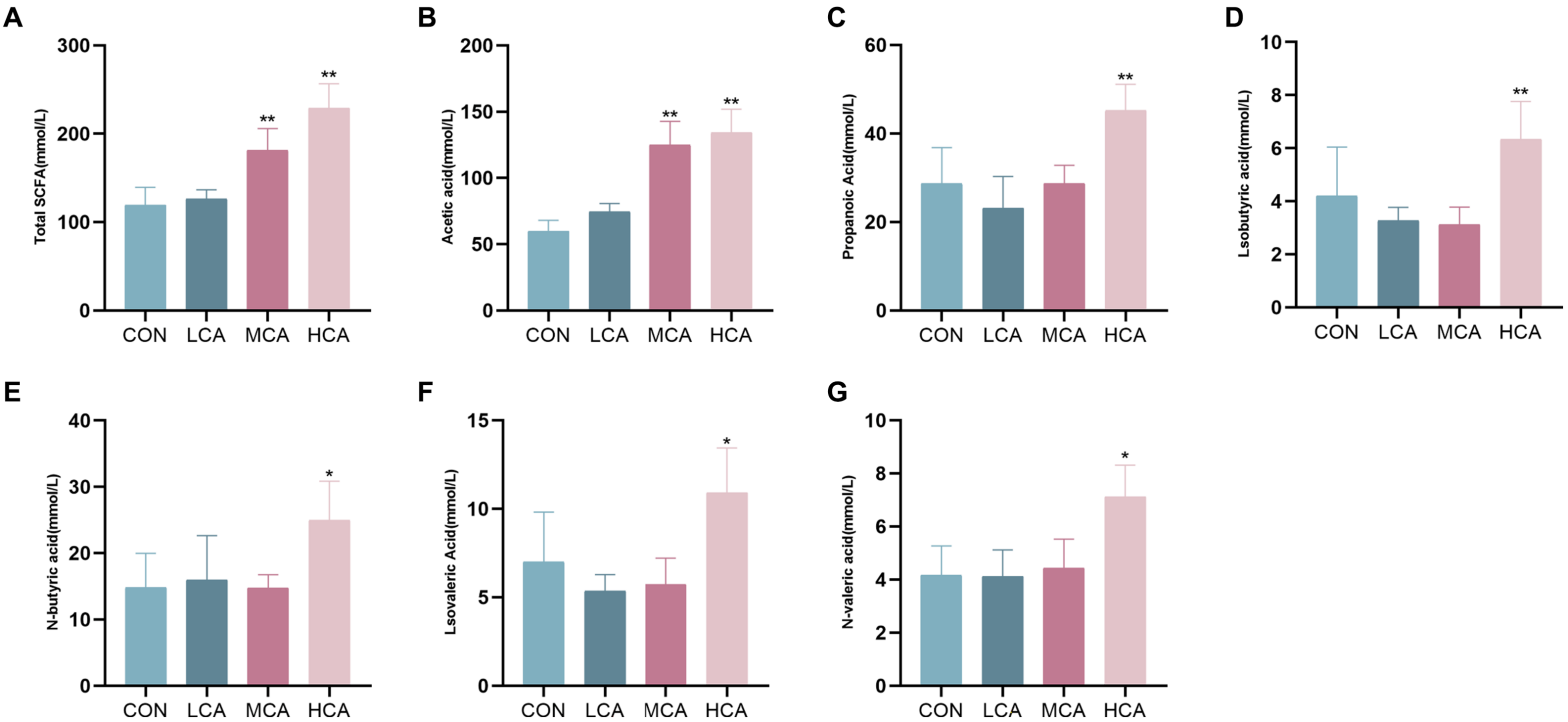
Effects of CA on the content of SCFAs in weaned piglets. **(A)** Fecal total SCFAs content. **(B)** Fecal Acetic acid content. **(C)** Fecal ProPionic acid. **(D)** Fecal Isobutyric acid. **(E)** Fecal N-butyric acid. **(F)** Isovaleric acid. **(G)** Fecal N-valeric acid. CON group (piglets fed basal diet). LCA group (piglets fed basal diet +0.5% CA). MCA group (piglets fed basal diet +1.0% CA). HCA group (piglets fed basal diet +1.5% CA). Mean ± SEM are shown (*n* = 6). ^*^Indicates statistical differences between groups: ^*^p < 0.05 indicates significant differences; ^**^p < 0.01 indicates highly significant differences.

The faecal samples showed the highest proportions of acetic acid and propionic acid. The MCA and HCA groups had significantly higher levels of total SCFAs and acetic acid compared to the CON group (*p* < 0.05). Furthermore, the HCA group exhibited significantly elevated levels of propionic acid, isobutyric acid, n-butyric acid, isovaleric acid, and n-valeric acid in comparison to the CON group (*p* < 0.05) (Figure 5).

#### 4.5.3 Correlation analysis between SCFAs and gut microbiota

**Figure 6:**
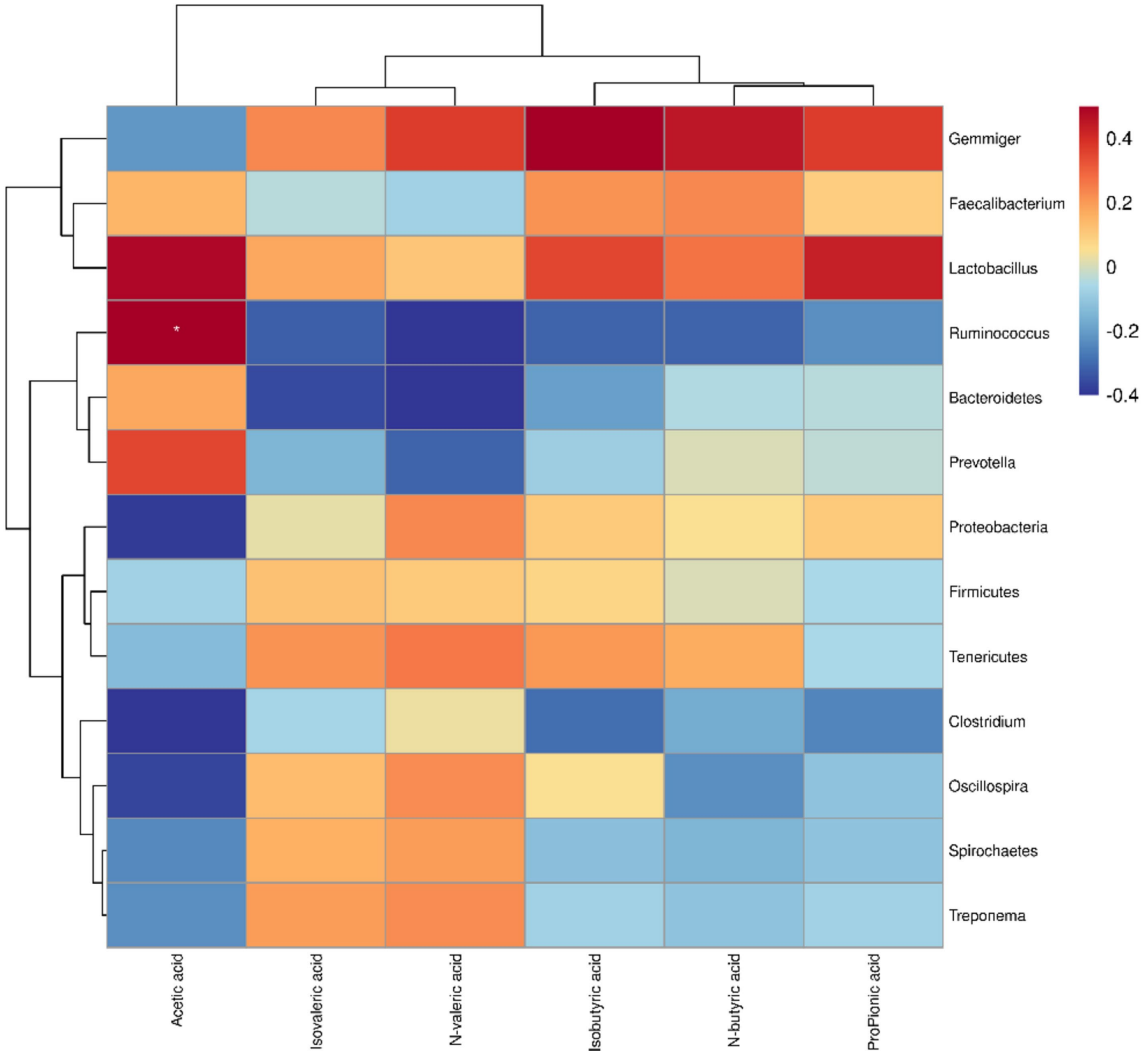
Spearman’s correlation between microbiota and SCFAs in weaned piglets. Red represents positive correlation and blue represents negative correlation. The intensity of the colours is proportional to the strength of the Spearman correlation. ^*^p < 0.05.

The results showed that Ruminococcus and Lactobacillus were short-chain fatty acid-producing bacteria (Figure 6). Spearman’s correlation analysis demonstrated a positive correlation between Ruminococcus and acetic acid, and between Lactobacillus and acetic and propionic acids (17, 18).

## 5 Discussion

Piglet weaning stress can cause increased intestinal permeability and decreased resistance (20), leading to piglets being prone to diarrhea within 1 week of weaning, high mortality, and poor juvenile growth and development, resulting in low productive performance in adulthood (8, 20). The intestinal tract plays a role in absorbing nutrients and warding off the invasion of pathogens, and the incidence of diarrhea is an important indicator for evaluating the degree of gut health (21). Improving the rearing environment and alleviating piglet weaning stress can effectively improve piglet health. Stress caused by weaning piglets may increase their energy and nutrient needs, thus reducing feed conversion efficiency (22). Furthermore some studies have reported that the addition of *Astragalus* polysaccharide, *Ginseng* polysaccharides, *Lycium* polysaccharides, *Clostridium butyricum*, *Quercetin*, and fermented *Codonopsis pilosulae* and *Astragalus membranaceus* to basic diets significantly increased the weight and reduced the incidence of diarrhea in piglets (23–27) These results are consistent with the results of the present study, indicating that the addition of CA to the basic diet can also improve the growth performance and feed conversion ratio of weaned piglets, and effectively reduce the incidence of piglet diarrhea. In this study, 1.5% CA was added to the basal diet, resulting in an increase in the diarrhea rate compared to the CON group. This increase may be attributed to the high dose of CA, which could lead to adverse reactions in the digestive system of piglets. Such reactions may include irritation of the gastrointestinal tract, thereby affecting the digestive and absorptive functions of the animals and ultimately causing diarrhea. Some studies have suggested that high doses of fermented herbs can effectively reduce diarrhea rates (28). However, the findings of this study indicate a different outcome when CA was used at the specified concentration. Deng et al. emphasized the importance of protein as an essential nutrient for piglet growth. Nevertheless, excessive protein intake can trigger allergic reactions, disrupt intestinal barrier function, promote pathogen growth, and exacerbate piglet diarrhea (29). In addition, it was found that the addition of CA significantly increased piglet feed intake, and it was hypothesized that the underlying mechanism might be that *Codonopsis pilosulae* and *Astragali seu Hedysari* are rich in polysaccharides, which are aromatic in odor and palatable, and thus increase piglet feed intake (30).

Oxidative stress is a common problem in pig farming, leading to reduced levels of animal welfare and quality of production. The weaning of piglets shows a dysfunction of the intestinal tract, increasing the level of free radicals and inhibiting the antioxidant system, resulting in a decrease in the antioxidant capacity of the organism (31). The antioxidant capacity of the body is commonly assessed by SOD, GSH-PX, T-AOC and MDA levels. MDA levels reflect the imbalanced state of the antioxidant defence system in piglets (32). SOD is widely found in organisms and is capable of scavenging superoxide anion radicals in organisms (33). GSH-PX is an important peroxidative catabolic enzyme in organisms, and the magnitude of its activity directly reflects the level of organismal Se (34). In addition, MDA levels are often considered representative indicators of lipid peroxidation (35). T-AOC levels represent the redox state *in vivo* (36). It was found that supplementation with *Codonopsis pilosulae* and *Astragalus membranaceus* may alleviate the effects of weaning stress by improving the antioxidant levels of weaned piglets and maintaining intestinal health (37). Crushed *Codonopsis pilosulae* and *Astragali seu Hedysari* were added to the basal diet, which elevated GSH-Px、CAT and SOD levels, and decreased MDA levels in the serum of green laying hens (38). In this study, the addition of CA to the basic diet significantly improved serum T-AOC, SOD, GSH-Px and MDA levels. These results suggest that the addition of CA to diets may improve the antioxidant capacity of weaned piglets by counteracting oxidative stress.

Weaning-induced inflammation may lead to slow growth of piglets in pig production. In this study, CA improves serum IgA, IgG, and IgM levels in weaned piglets and enhances body immunity. Immunoglobulins, including IgA, IgG, and IgM, protect the organism by removing foreign antigens and have the function of strengthening the body’s immunity, preventing the invasion of viruses and bacteria, neutralizing toxins, and killing tumor cells (39). Polysaccharides, the main active components of *Codonopsis pilosulae* and *Astragalus membranaceus*, bind to a variety of receptors on the surface of immune cells and activate the relevant signaling pathways, thereby regulating the body’s immune system (40). However, when the body undergoes an excessive immune response, a portion of the body’s energy and nutrition will be used for the excessive immune response (41). Therefore, the addition of CA to the feed can enhance the immune ability of weaned piglets and keep the immunoglobulins and anti-inflammatory cytokines at the appropriate level, so as to achieve the suppression of various adverse reactions after weaning.

The health of the body and intestinal flora are inextricably linked, intestinal flora dysbiosis makes the intestinal mucosa damaged, intestinal permeability increases, making it easy for intestinal diseases to occur (42). In this study, it was found that microbial composition and structure differed between the experimental groups, with the phylum Firmicutes and Bacteroidetes accounting for the predominant phyla in the faeces of weaned piglets and the abundance of the beneficial bacteria Firmicutes, Bacteroidetes, Lactobacillus, Faecalibacterium, Clostridiaceae and Prevotella being all up-regulated (20). Lan et al. showed that feeding *Astragalus membranaceus*, *Codonopsis pilosula* and *allicin* mixture could change the faecal microbiota of finishing pigs by increasing the number of Lactobacillus spp. and decreasing the number of *Escherichia coli* to improve the microbial community, and that bacterial microorganisms in the pig’s cecum were predominantly in the Firmicutes and Bacteroidetes (12, 43). Faecalibacterium are considered to be probiotics that protect the digestive system from intestinal pathogens (44). Bacteroidetes and Firmicutes produce nutrients for use by the organism though degrading plant fibers (45). Spirochaetes is strongly associated with inflammatory diseases of the intestinal tract and diarrhea in the body. Intestinal flora can digest food, synthesize essential vitamins, stimulate and regulate the immune system, and eliminate pathogens, etc. A balanced intestinal flora can help prevent disease (46). CA is beneficial in promoting the growth of beneficial intestinal bacteria (Firmicutes, Bacteroidetes, Clostridium, Lactobacillus, Faecalibacterium) and inhibiting the growth of harmful bacteria(Spirochaetes), maintaining the balance of intestinal microbial community and improving intestinal health.

SCFAs, the main metabolites produced by gut microbes, protect intestinal health by reducing inflammation, maintaining intestinal integrity, and modulating the immune response. They are also involved in inhibiting histone deacetylases and activating G-coupled receptors (4, 7, 47). Acetic acid and propionic acid are absorbed by intestinal epithelial cells to reach the peripheral blood and liver where they are utilized for energy and participate in glycogen synthesis (48). Additionally, SCFAs inhibit pathogen reproduction by lowering the host’s intestinal pH and enhancing the richness of the intestinal microbiota. Astragalus fiber has been shown to improve the SCFAs of weaned piglets and regulate their microbial community, while a related study demonstrated a decrease in the abundance of Bacteroidetes and Spirochaetes in the fecal microbial community of piglets with diarrhea (49, 50). Furthermore, Clostridium has been strongly correlated with acetic acid and total SCFAs (51). Finally, CA has been found to promote the abundance of Ruminococcacea and Lactobacillus producing acetic acid and propionic acid, thereby increasing the content of SCFAs in weaned piglets and potentially playing a positive role in host immunity and health by modulating the intestinal microbiota to promote SCFAs production.

## 6 Conclusion

The addition of 1.0% of CA to the basal diet improved growth performance and reduced the incidence of diarrhea in weaned piglets. This optimal additive level may have contributed to the beneficial effects, which can be explained by the improved antioxidant capacity, immune response, gut microbiota and SCFAs. These results suggest that CA can serve as an alternative to antibiotics in weaned piglet feed development.

## Data Availability

The data presented in the study are deposited in the NCBI repository, accession number PRJNA1144829. Access via the following link: https://www.ncbi.nlm.nih.gov/sra/PRJNA1144829.

## References

[1] Sun T Miao H Zhang C Wang Y Liu S Jiao P. Effect of dietary *Bacillus coagulans* on the performance and intestinal microbiota of weaned piglets. Animal. (2022) 16:100561. doi: 10.1016/j.animal.2022.100561, PMID: 35716416

[2] Zhang F Chen M Liu X Ji X Li S Jin E. New insights into the unfolded protein response (UPR)-anterior gradient 2 (AGR2) pathway in the regulation of intestinal barrier function in weaned piglets. Anim Nutr. (2023) 15:225–32. doi: 10.1016/j.aninu.2023.08.007, PMID: 38033605 PMC10685161

[3] Tang X Xiong K Fang R Li M. Weaning stress and intestinal health of piglets: a review. Front Immunol. (2022) 13:1042778. doi: 10.3389/fimmu.2022.1042778, PMID: 36505434 PMC9730250

[4] Liu Y Azad MAK Ding S Zhu Q Blachier F Yu Z. Dietary bile acid supplementation in weaned piglets with intrauterine growth retardation improves colonic microbiota, metabolic activity, and epithelial function. J Animal Sci Biotechnol. (2023) 14:99. doi: 10.1186/s40104-023-00897-2, PMID: 37438768 PMC10339644

[5] Bai X Yan X Xie L Hu X Lin X Wu C. Effects of pre-slaughter stressor and feeding preventative Chinese medicinal herbs on glycolysis and oxidative stability in pigs. Anim Sci J. (2016) 87:1028–33. doi: 10.1111/asj.12537, PMID: 26497952

[6] Li J. Current status and prospects for in-feed antibiotics in the different stages of pork production – a review. Asian Australas J Anim Sci. (2017) 30:1667–73. doi: 10.5713/ajas.17.0418, PMID: 28823126 PMC5666167

[7] Wu Y Zhao JF Xu C Ma N He T Zhao JS. Progress towards pig nutrition in the last 27 years. J Sci Food Agric. (2020) 100:5102–10. doi: 10.1002/jsfa.9095, PMID: 29691867

[8] Wei YY Zang YN Fan YM Ga Y Wang HR Han JC. Comparative study on piglet weaning stress syndrome and diarrhea type irritable bowel syndrome with liver stagnation and spleen deficiency syndrome. Acta Neuropharmacol. (2022) 12:44–51.

[9] Chi YK Liu XG Xiong KN Chen H Xiao H. Research Progress in application of Chinese herbal medicine feed additives. Chin Wild Plant Resour. (2020) 39:57–63.

[10] Li YP Li HH Wang Q Qiao JY Yao SN. Effects of *Clostridium butyricum* on the intestinal health of piglets. China Feed. (2020) 39:13–6.

[11] Hao YY Nie CX Wu XW Liu C Hao XL. Research progress of *Codonopsis pilosula* polysaccharide and its structure modification on immune regulation. China Med Her. (2018) 15:25–8.

[12] Lan RX Park JW Lee DW Kim IH. Effects of Astragalus membranaceus, Codonopsis pilosula and allicin mixture on growth performance, nutrient digestibility, faecal microbial shedding, immune response and meat quality in finishing pigs. J Anim Physiol Anim Nutr (Berl). (2017) 101:1122–9. doi: 10.1111/jpn.12625 27868250

[13] Ma JQ Du XF Zheng LM. Codonopsis polysaccharide inhibits the expression of inflammatory and fibrotic components in high glucose-induced human renal tubular epithelial cells. Chin J Integr Tradit West Nephrol. (2022) 23:681–3.

[14] Ma YC Hu JH Wu WX Duan Y Fan CC Feng TT. Research Progress on chemical constituents and pharmacological effects of Radix Astragali. Acta Chin Med Pharmacol. (2022) 50:92–5.

[15] Xie WC Song LY Wang XY Xu YG Liu ZS Zhao DF. A bovine lactoferricin-lactoferrampin-encoding *Lactobacillus reuteri* CO21 regulates the intestinal mucosal immunity and enhances the protection of piglets against enterotoxigenic *Escherichia coli* K88 challenge. Gut Microbes. (2021) 13:1956281. doi: 10.1080/19490976.2021.1956281, PMID: 34369287 PMC8354667

[16] Jiang KC Cheng Y Jiao SY Wang J Tuo XH Han B. Rapid determination of 6 short-chain fatty acids in feces by gas chromatography. ModernPreventive Medicine. (2020) 47:686–689+711.

[17] Canfora EE Jocken JW Blaak EE. Short-chain fatty acids in control of body weight and insulin sensitivity. Nat Rev Endocrinol. (2015) 11:577–91. doi: 10.1038/nrendo.2015.128 26260141

[18] Zhang XM Li M Chai YH Zhou J Lei H. Research progress on the correlation between intestinal microbiota short chain fatty acids and pulmonary tuberculosis. Chin J Antituberculosis. (2023) 45:699–706.

[19] Yu GH Jiang JY Wang XT Zhang HJ Geng M Song CY. Effects of piglets weaning stress on intestine development and histomorphology. Chin J Vet Med. (2018) 54:23-26+29.

[20] Campbell JM Crenshaw JD Polo J. The biological stress of early weaned piglets. J Anim Sci Biotechnol. (2013) 4:19. doi: 10.1186/2049-1891-4-19, PMID: 23631414 PMC3651348

[21] Kong Q Zhang W An M Kulyar MF-E-A Shang Z Tan Z. Characterization of bacterial microbiota composition in healthy and diarrheal early-weaned Tibetan piglets. Front Vet Sci. (2022) 9:799862. doi: 10.3389/fvets.2022.799862, PMID: 35280137 PMC8905297

[22] Patience JF Rossoni-Serão MC Gutiérrez NA. A review of feed efficiency in swine: biology and application. J Anim Sci Biotechnol. (2015) 6:33. doi: 10.1186/s40104-015-0031-2 26251721 PMC4527244

[23] Liang J Kou S Chen C Raza SHA Wang S Ma X. Effects of *Clostridium butyricum* on growth performance, metabonomics and intestinal microbial differences of weaned piglets. BMC Microbiol. (2021) 21:85. doi: 10.1186/s12866-021-02143-z, PMID: 33752593 PMC7983215

[24] Xu B Qin W Xu Y Yang W Chen Y Huang J. Dietary quercetin supplementation attenuates diarrhea and intestinal damage by regulating gut microbiota in weanling piglets. Oxid Med Cell Longev. (2021):6221012. doi: 10.1155/2021/6221012 34950418 PMC8689231

[25] Yang CM Han QJ Wang KL Xu YL Lan JH Cao GT. Astragalus and ginseng polysaccharides improve developmental, intestinal morphological, and immune functional characters of weaned piglets. Front Physiol. (2019) 10:418. doi: 10.3389/fphys.2019.00418, PMID: 31031640 PMC6473041

[26] Yin YX Wang F Yang M Tan B Yin YL Chen J. *Lycium barbarum* polysaccharides as antibiotic substitutes improve growth performance, serum immunity, antioxidant status, and intestinal health for weaned piglets. Front Microbiol. (2021) 12:819993. doi: 10.3389/fmicb.2021.819993 35281314 PMC8914510

[27] Wei HF Liang ZT Guo JL Hu YL. Effects of fermented Astragalus membranaceus and *Codonopsis pilosula* on growth performance and serum biochemical indexes of weaned piglets. Chin J Anim Sci. (2020) 56:132–4.

[28] Chen G Li ZQ Liu SL Tang T Chen QH Yan ZM. Fermented Chinese herbal medicine promoted growth performance, intestinal health, and regulated bacterial microbiota of weaned piglets. Animals (Basel). (2023) 13:476. doi: 10.3390/ani13030476, PMID: 36766365 PMC9913397

[29] Han XB Hu XD Jin W Liu G. Dietary nutrition, intestinal microbiota dysbiosis and post-weaning diarrhea in piglets. Anim Nutr. (2024) 17:188–207. doi: 10.1016/j.aninu.2023.12.010, PMID: 38800735 PMC11126776

[30] Aebisher D Cichonski J Szpyrka E Masjonis S Chrzanowski G. Essential oils of seven Lamiaceae plants and their antioxidant capacity. Molecules. (2021) 26:3793. doi: 10.3390/molecules26133793 34206525 PMC8270304

[31] Yin J Wu MM Xiao H Ren WK Duan JL Yang G. Development of an antioxidant system after early weaning in piglets. J Anim Sci. (2014) 92:612–9. doi: 10.2527/jas.2013-6986, PMID: 24352957

[32] Zhu H Jia Z Misra H Li YR. Oxidative stress and redox signaling mechanisms of alcoholic liver disease: updated experimental and clinical evidence. J Dig Dis. (2012) 13:133–42. doi: 10.1111/j.1751-2980.2011.00569.x, PMID: 22356308 PMC3297983

[33] Xu J Xu C Chen X Cai X Yang S Sheng Y. Regulation of an antioxidant blend on intestinal redox status and major microbiota in early weaned piglets. Nutrition. (2014) 30:584–9. doi: 10.1016/j.nut.2013.10.018, PMID: 24698350

[34] Handy DE Loscalzo J. The role of glutathione peroxidase-1 in health and disease. Free Radic Biol Med. (2022) 188:146–61. doi: 10.1016/j.freeradbiomed.2022.06.004 35691509 PMC9586416

[35] Jiang W-D Wen H-L Liu Y Jiang J Wu P Zhao J. Enhanced muscle nutrient content and flesh quality, resulting from tryptophan, is associated with anti-oxidative damage referred to the Nrf2 and TOR signalling factors in young grass carp (*Ctenopharyngodon idella*): avoid tryptophan deficiency or excess. Food Chem. (2016) 199:210–9. doi: 10.1016/j.foodchem.2015.12.003 26775963

[36] Benzie IF Strain JJ. The ferric reducing ability of plasma (FRAP) as a measure of “antioxidant power”: the FRAP assay. Anal Biochem. (1996) 239:70–6. doi: 10.1006/abio.1996.0292, PMID: 8660627

[37] Tao J Meng L Wei HX Li L Zhang SQ. Effects of different Chinese herbal medicines on growth performance, immunity, stress resistance and antioxidant capacity of weaned piglets. Heilongjiang Anim Sci Vet Med. (2020):112–7.

[38] He LQ Qu XY Wei YH Wang JM Chang CR Xiao JX. Effects of selenium yeast and tea polyphenols and their interaction on production performance and antioxidant capacity of hens laying green shelled egg. J Hunan Agric Univ (Nat Sci). (2012) 38:417–21.

[39] Planchais C Mouquet H. Easy pan-detection of human IgA immunoglobulins. J Immunol Methods. (2020) 484–485:112833. doi: 10.1016/j.jim.2020.112833 32771390

[40] Ren ZH Yuan W Deng HD Deng JL Dan QX Jin HT. Effects of antibacterial peptide on cellular immunity in weaned piglets. J Anim Sci. (2015) 93:127–34. doi: 10.2527/jas.2014-7933 25403191

[41] Niu X Ding Y Chen S Gooneratne R Ju X. Effect of immune stress on growth performance and immune functions of livestock: mechanisms and prevention. Animals (Basel). (2022) 12:909. doi: 10.3390/ani12070909, PMID: 35405897 PMC8996973

[42] Azad MAK Gao J Ma J Li T Tan B Huang X. Opportunities of prebiotics for the intestinal health of monogastric animals. Anim Nutr. (2020) 6:379–88. doi: 10.1016/j.aninu.2020.08.001, PMID: 33364453 PMC7750794

[43] Jandhyala SM Talukdar R Subramanyam C Vuyyuru H Sasikala M Nageshwar Reddy D. Role of the normal gut microbiota. World J Gastroenterol. (2015) 21:8787–803. doi: 10.3748/wjg.v21.i29.8787, PMID: 26269668 PMC4528021

[44] Michalak L Gaby JC Lagos L La Rosa SL Hvidsten TR Tétard-Jones C. Microbiota-directed fibre activates both targeted and secondary metabolic shifts in the distal gut. Nat Commun. (2020) 11:5773. doi: 10.1038/s41467-020-19585-0, PMID: 33188211 PMC7666174

[45] Borbón-García A Reyes A Vives-Flórez M Caballero S. Captivity shapes the gut microbiota of Andean bears: insights into health surveillance. Front Microbiol. (2017) 8:1316. doi: 10.3389/fmicb.2017.01316, PMID: 28751883 PMC5507997

[46] Konstantinov SR Awati AA Williams BA Miller BG Jones P Stokes CR. Post-natal development of the porcine microbiota composition and activities. Environ Microbiol. (2006) 8:1191–9. doi: 10.1111/j.1462-2920.2006.01009.x, PMID: 16817927

[47] Koh A De Vadder F Kovatcheva-Datchary P Bäckhed F. From dietary fiber to host physiology: short-chain fatty acids as key bacterial metabolites. Cell. (2016) 165:1332–45. doi: 10.1016/j.cell.2016.05.041, PMID: 27259147

[48] den Besten G van Eunen K Groen AK Venema K Reijngoud D-J Bakker BM. The role of short-chain fatty acids in the interplay between diet, gut microbiota, and host energy metabolism. J Lipid Res. (2013) 54:2325–40. doi: 10.1194/jlr.R036012, PMID: 23821742 PMC3735932

[49] Che D Adams S Wei C Gui-Xin Q Atiba EM Hailong J. Effects of Astragalus membranaceus fiber on growth performance, nutrient digestibility, microbial composition, VFA production, gut pH, and immunity of weaned pigs. Microbiology. (2019) 8:e00712. doi: 10.1002/mbo3.712 PMC6528644 30117299

[50] Qi M Cao Z Shang P Zhang H Hussain R Mehmood K. Comparative analysis of fecal microbiota composition diversity in Tibetan piglets suffering from diarrheagenic *Escherichia coli* (DEC). Microb Pathog. (2021) 158:105106. doi: 10.1016/j.micpath.2021.105106, PMID: 34311015

[51] Li H Ma L Li Z Yin J Tan B Chen J. Evolution of the gut microbiota and its fermentation characteristics of Ningxiang pigs at the young stage. Animals (Basel). (2021) 11:638. doi: 10.3390/ani11030638 33673705 PMC7997423

